# Targeting LMW‐PTP to sensitize melanoma cancer cells toward chemo‐ and radiotherapy

**DOI:** 10.1002/cam4.1435

**Published:** 2018-03-24

**Authors:** Giulia Lori, Paolo Paoli, Anna Caselli, Paolo Cirri, Riccardo Marzocchini, Monica Mangoni, Cinzia Talamonti, Lorenzo Livi, Giovanni Raugei

**Affiliations:** ^1^ Department of Experimental and Clinical Biomedical Sciences University of Florence Florence Italy

**Keywords:** anticancer therapy, apoptosis, chemoresistance, LMW‐PTP, sensitizing agent

## Abstract

Tumor resistance to apoptosis is one the main causes of anticancer treatment failure. Previous studies showed that LMW‐PTP overexpression enhances resistance of cancer cells to traditional anticancer drugs. Today, the role of LMW‐PTP in inducing resistance to apoptosis in melanoma cells remains to be elucidated. Experimental setting include MTT assay, Annexin V/Pi method, and colony assay to assess whether silencing of LMW‐PTP improves the sensitivity of A375 to dacarbazine, 5‐FU, and radiotherapy. Pharmacological targeting of LMW‐PTP was obtained using Morin, a LMW‐PTP inhibitor. The ability of Morin to improve the effectiveness of anticancer drugs and radiotherapy was also studied. Moreover, PC3 cells were used as an alternative cellular model to confirm the data obtained with melanoma cells. We found that LMW‐PTP silencing improves the effectiveness of dacarbazine, 5‐FU, and radiotherapy. Identical results were obtained in vivo when Morin was used to target LMW‐PTP. We demonstrated that Morin synergizes with dacarbazine, improving its cytotoxic activity. However, we showed that the combined treatment, Morin‐anticancer drug, does not affect the viability of noncancerous cells. Knockdown of LMW‐PTP sensitizes also PC3 cells to docetaxel and radiotherapy. In conclusion, we showed that LMW‐PTP targeting improves effectiveness of anticancer drugs used for treatment of melanoma. Moreover, our results suggest that Morin could be used as adjuvant to improve the outcome of patients affected by metastatic melanoma.

## Introduction

Malignant melanoma is one of the most aggressive types of skin cancer, showing a high metastatic potential and mortality 1. In fact, even if melanoma represents only a small proportion of skin cancer (4%), it accounts for 80% of skin cancer‐related mortality 2. The main risk factors in the development of melanoma include intense exposure to ultraviolet light (UV), familial history of melanoma, or nonmelanoma skin cancer 3, 4. Different therapeutically protocols are used to treat melanoma, depending on the stage of pathology. When melanoma is diagnosed in the early stages, surgical ablation of the tumor is recommended; in other cases, surgery is associated with different types of adjuvant therapies, or with radiotherapy. However, in the case of metastatic melanoma, chemotherapy is often the only therapeutical option. Alkylating agents such as dacabarzine, temozolomide, or fotemustine are the most recommended drugs, even if these generally show a modest efficacy and induce heavy side effects in the majority of patients 1, 5.

Intrinsic resistance to apoptosis of melanoma cells is one of the main causes of anticancer therapy failure. Many factors contribute to the deregulation of the apoptotic mechanism, including the lost or mutation of the P53 gene, the over‐activation of the survival pathway, overexpression of anti‐apoptotic proteins and of drug extrusion pumps, or DNA repairing proteins 6. There is a substantial agreement about the fact that the development of new strategies useful to overcome intrinsic drug resistance of melanoma cells could have a significant impact on the survival rate of patients affected by this kind of cancer.

In the last decades, compelling evidence suggested that LMW‐PTP could have an important role in modulating a response of cancer cells to genotoxic insults. LMW‐PTP can be considered an early marker of carcinogenesis. Its expression is increased in preneoplastic lesions of colon from rats treated with tumor inducing agents 7. Further studies showed that LMW‐PTP is overexpressed in different cancers, including colon cancer and neuroblastoma, and that its expression is correlated with a worse prognosis and a reduced survival rate 8. Overexpression of LMW‐PTP in NIH‐3T3 cells is sufficient to induce neoplastic transformation, and LMW‐PTP‐transfected NIH3T3 fibroblasts generate large tumors once engrafted in nude mice 9. In addition, a study carried out with 481 male patients suffering of prostate cancer highlighted that LMW‐PTP is over‐expressed in prostate cancer cells, and its expression correlates with a worse prognosis and with disease recurrence 10.

More recently, independent studies demonstrated that LMW‐PTP is involved in the regulation of apoptosis and in the acquisition of drug resistance. Ferreira and colleagues demonstrate that over‐expression of LMW‐PTP confers resistance to vincristine in leukemic cells 11. Similar results have been obtained by a study focused on colorectal cancer, showing that the LMW‐PTP over‐expression mediates the malignant potential of cancer cells, inducing drug resistance and enhanced cell motility and invasiveness 12. Together, these evidence suggest that LMW‐PTP is a key player in sustained tumor growth and resistance of cancer cells toward traditional anticancer therapies.

This study aimed to define the role of LMW‐PTP in inducing resistance of melanoma cells to chemo‐ and radiotherapy. We show that melanoma cells express high LMW‐PTP levels in comparison with normal fibroblasts. Moreover, using RNA interference technology, we demonstrate that LMW‐PTP knockdown increases the sensitivity of melanoma cells to dacarbazine, 5‐FU, and radiotherapy and impairs the clonogenic ability of cancer cells. Furthermore, we show that treating melanoma cells with Morin, a natural polyphenol that in vitro inhibits both LMW‐PTP isoforms, we can reproduce all effects induced by RNA interference. Interestingly, we find that Morin is active against cancer cells that overexpress LMW‐PTP, but is ineffective against noncancer cells that express low LMW‐PTP levels. Finally, we demonstrate that LMW‐PTP downregulation sensitize PC3 cells toward docetaxel, suggesting that the role of LMW‐PTP as a negative regulator of apoptosis is not limited to melanoma cells. Taken together, these findings confirm the role of LMW‐PTP in inducing resistance toward traditional anticancer therapy, and open new opportunities for designing innovative therapeutic approaches based on the use of specific LMW‐PTP inhibitors as chemosensitizing agents for the treatment of melanoma and other type of chemo‐ or radio‐resistant tumors.

## Material and Methods

### Cell culture and transfections

A375 melanoma cells were purchased from ATCC (Manassas, VA) and cultured in Dulbecco's modified Eagle's medium (DMEM; Sigma Aldrich, St. Louis, MO), supplemented with 10% Fetal Bovine Serum, 100 U/mL penicillin, 100 mg/mL streptomycin (both from Euroclone), and propagated at 37°C in a 5% CO_2_ humidified atmosphere. A375 cells (100,000 cells/mL) were grown for 24 h and then transiently transfected with LMW‐PTP siRNA (Target sequence CCCATAGTGCACACTTGTATA), using the Hiperfect Transfection Reagent (Qiagen Italia, Via Filippo Sassetti, 16, 20124 Milano, Italy) according to the manufacturer's instructions. Briefly, the cells were transfected for 24, 48, or 72 h with siRNA at a final concentration of 20 nmol/L. To test the specificity of LMW‐PTP transfection, control cells were transfected with a Scramble Sequence (AllStars Negative Control siRNA; at a final concentration of 20 nmol/L; Qiagen). Western blotting assessed the efficiency of transfection.

### Enzymatic assay

Enzymatic assays were carried out using purified recombinant human LMW‐PTPs purified as previously described 13. Phosphatase assay was carried out at 37°C using p‐nitrophenylphosphate as substrate dissolved in a solution containing 0.075 mol/L of *β*,*β*‐dimethylglutarate buffer (pH 7.0), 1 mmol/L EDTA, and 5 mmol/L dithiothreitol. All reactions were initiated by the addition of the enzyme in the solutions and stopped by adding 4 mL of 1 mol/L KOH. The amount of p‐nitrophenolate released was determined using a spectrophotometer, reading the absorbance of samples at 400 nm (*ε *= 18,000/M*cm). Km and Vmax were determined by measuring the initial hydrolysis rate of enzyme at different substrate concentrations. Experimental data were analyzed using the Michaelis–Menten equation and a nonlinear fitting program (Fig‐Sys BIOSOFT Great Shelford Cambridge CB22 5WQ, United Kingdom). Inhibition constants were determined by measuring initial hydrolysis rates at differing substrate and inhibitor concentrations. The apparent Km values measured at the various inhibitor concentrations were plotted against concentration of the inhibitor to calculate the Ki values. All tests were carried out in triplicate.

### Evaluation of synergism between Morin and dacarbazine

Synergism between Morin and dacarbazine was determined using method proposed by Ting‐Chao Chou 14. Data obtained were analyzed using Compusyn computer program 15.

### Western Blotting

Cells were lysed on ice in 1× Laemli Buffer (0.5 mol/L Tris–HCl pH 6.8, 10% SDS, 20% glycerol, *β*‐mercaptoethanol, 0.1% bromophenol blue), and samples were boiled for 10 min. Cell extracts were resolved by SDS‐PAGE and transferred to PVDF membranes (Bio‐Rad Laboratories S.r.l. Via Cellini, 18/A 20090 Segrate (MI), Italy). Membranes were incubated overnight at 4°C with the appropriate primary antibody: rabbit polyclonal anti‐LMW‐PTP antibodies were produced in our laboratory, Bcl‐2, Bim, Caspase3, Actin were obtained from Santa Cruz Biotechnology, Inc. (Bergheimer Str. 89‐2 69115, Heidelberg Germany). After washing in TPBS‐Tween‐20 (0.1%), membranes were incubated with the appropriate horseradish peroxidase‐conjugated secondary antibodies (Santa Cruz Biotechnology) for 1 h. Proteins were detected using Clarity Western ECL (Bio‐Rad) by UVP.

### Cell viability assay

2 × 10^4^ cells were seeded in 24‐well plate: after 24, 48, or 72 h 5 mmol/L MTT (3‐(4,5‐Dimethylthiazol‐ 2‐yl)‐2,5‐diphenyltetrazolium bromide was added and incubated for 2 h at 37°C. Cells were resuspended in 200 *μ*L of dimethyl sulfoxide: wavelength measuring was performed at 595 nm using a spectrophotometer.

### Apoptosis evaluation

Apoptosis was determined using Annexin‐V‐FLUOS Staining kit from Roche according to manufacturer's instructions. 1 × 10^6^ cells were detached with Acutase, washed in PBS, and centrifuged at 1,300 g for 5 min. Cell pellet was resuspended in 100 *μ*L of Annexin‐V‐FLUOS labeling solution and incubated for 10–15 min at room temperature in the dark. Five hundred microliters of incubation buffer was added, and cells were analyzed by flow cytometry BDFACS Canto.

### Colony Formation Assay

Briefly, cells were seeded in six‐well plate and treated with Morin or trasfected with siRNA at the indicated concentration. After 24 h, the cells were detached and re‐plated 1000 cells/well and incubated at 37°C for 10 days. Subsequently, cells were fixed and stained with a solution containing 1% crystal violet and 20% methanol. Colonies were counted and photographed using NIKON Digital Sight. Plating efficiency and surviving factor were extrapolated using the following formulas:

$$\text{PE}=\left(\frac{\text{no of colonies}}{\text{no of cells seeded}}\right)\times 100,$$

$$\text{SF}=\left(\frac{\text{no of colonies}}{\text{no of cells seeded}}\right)\times \text{PE},$$

All data obtained were normalized respect to control test.

### Adhesion test

A375 cells were seeded on 35‐mm Petri dishes. When cells reached appropriate density (70% confluence), these are washed with PBS and incubated in the presence of 0.05% trypsin solution. After 10 min, the dishes were washed with PBS containing 0.5 mL/mL of soybean trypsin inhibitor to stop proteolytic trypsin activity. Then, the cells were incubated for 5 min at 37°C with 0.5% crystal violet solution. Extensive washing was carried out using PBS to remove the excess of dye that had not penetrated into the cells. The releasing of the dye absorbed by cells was obtained by incubating cells with 0.1 mol/L citrate buffer, pH 4.2, for 1 h at 37°C. After this time lapse, the citrate solution was withdrawn, to allow the quantification of the dye released from cells using a spectrophotometer. All samples were read at 595 nm using a one‐cm pathlength cuvette.

### Gelatin zymography

Conditioned media were collected and subjected to electrophoresis on 7.5% PAGE gels containing 0.1% gelatin. After electrophoresis, the gel was washed twice with 2.5% Triton X‐100 and once with reaction buffer (50 mmol/L Tris–HCl, pH 7.5, 200 mmol/L NaCl, 5 mmol/L CaCl_2_). The gel was incubated overnight at 37°C with fresh reaction buffer. Then, the gel was stained with 0.25 Coomassie Brilliant Blue and destained (30% methanol and 10% acetic acid).

### Boyden chamber assay

Cell invasion was performed with 1 × 10^5^ cells on 8‐*μ*m‐pore Transwells (Corning, One Riverfront Plaza Corning, NY) coated with 50 *μ*g/cm^2^ of reconstituted matrigel as described in 16. Chemotaxis was evaluated by counting the cells that had migrated to the lower surface of the filters (six randomly chosen fields).

### Cell Irradiation

Cell cultures were irradiated with a 6MV X‐Ray beam delivered by a clinical linear accelerator ELEKTA Synergy Beam Modulator. Single doses of 0–4 Gy were delivered to the cell cultures seeded in well plates. To provide a uniform irradiation, the dishes were embedded in water equivalent medium.

### Statistical analysis

Statistical analysis of the data was performed by unpaired Student's *t*‐test for pairwise comparison of groups unpaired *t*‐test. All data were expressed as the mean ± SEM A *P*‐value <0.05 was considered statistically significant. Statistical analysis was carried out on biological replicates, as indicated in the figure legends.

## Results

### Detection of LMW‐PTP in melanoma cells

The expression of LMW‐PTP in melanoma cells was evaluated by immunoblot, using antibodies able to recognize both LMW‐PTP isoforms. A375 cells, likewise PC3, HT29, HCT8, and SHS5Y cancer cells, express significantly higher levels of LMW‐PTP in comparison with Human Dermal Fibroblasts, which represent an example of noncancerous cells (Figure S1A and B). To confirm the clinical significance of LMW‐PTP in melanoma, we analyzed data obtained from the Oncomine database 17. We found that LMW‐PTP expression is significantly higher in melanoma cells than in normal skin cells (*P = 1.72186E‐5) (Figure S1C). This last finding reinforces the hypothesis that LMW‐PTP is essential to ensure melanoma cells growth and survival.

### LMW‐PTP knockdown impairs invasiveness, resistance to cytotoxic drugs, to radiotherapy, and self‐renewal ability of melanoma cells

To clarify the role of LMW‐PTP in melanoma cells, we analyzed the behavior of A375 cells silenced for LMW‐PTP (Figure S1D and E). We found that siRNA‐transfected cells acquire a spindle‐shape morphology and develop several plasma membrane protrusions (Figure S1F). Moreover, these adhere more strongly to plastic well (Figure S1G), express lower levels of MMP‐9 (Figure S1H), and are less invasive than control cells (Figure S1I). Together, these findings suggest that LMW‐PTP knockdown contributes to reprogram A375 cells toward a less aggressive phenotype.

**Figure 1:**
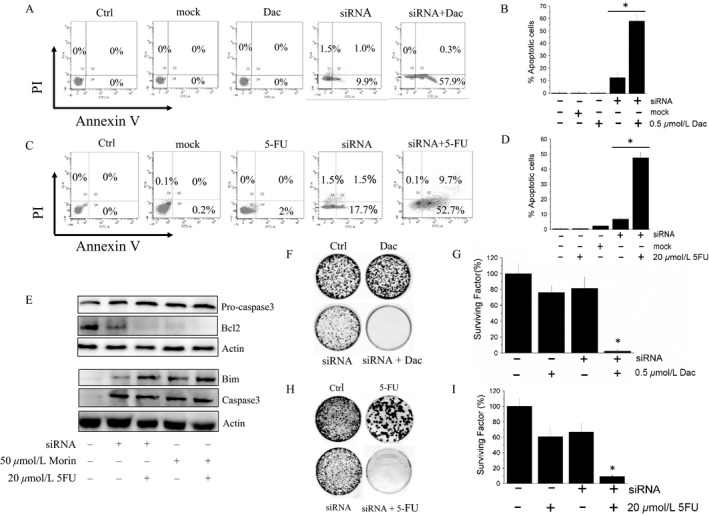
LMW‐PTP knockdown sensitizes A375 cells to apoptosis and impairs their self‐renewal capability. (A–D) detection of apoptosis in melanoma cells silenced for LMW‐PTP and treated with dacarbazine, and 5‐FU. (E) detection of apoptotic markers in melanoma cells silenced for LMW‐PTP or treated with Morin. (F and I), colony assay carried out with parental and A375 silenced cells. All data reported in the figure represent the mean values ± SEM (*n* = 3). *P < 0.05

Aggressiveness and drug resistance are often closely related features 18, 19, 20. This finding prompted us to evaluate whether LMW‐PTP silencing could improve sensitivity of A375 cells to apoptosis. We observed that the fraction of apoptotic cells is slightly higher in silenced cells with respect to control cells and reaches 50–60% in silenced cells treated with dacarbazine or 5‐FU. Conversely, we found that treatment with drugs does not impair the vitality of parental cells (Fig. 1A–D). In accord with this evidence, Western blot analysis showed that LMW‐PTP knockdown is associated with a drastic change of expression levels of Bcl2 and Bim, and with the activation of caspase 3 (Fig. 1E). Finally, colony assay shows that combining gene silencing with drug treatment is possible to inhibit clonogenic activity of melanoma cells (Fig. 1F–I). Together, these results suggested that LMW‐PTP knockdown contributes to impair melanoma cells aggressiveness, to sensitize melanoma cells toward apoptosis, and to inhibit their self‐renewal ability.

### LMW‐PTP knockdown increases sensitivity of A375 cells to radiotherapy

**Figure 2:**
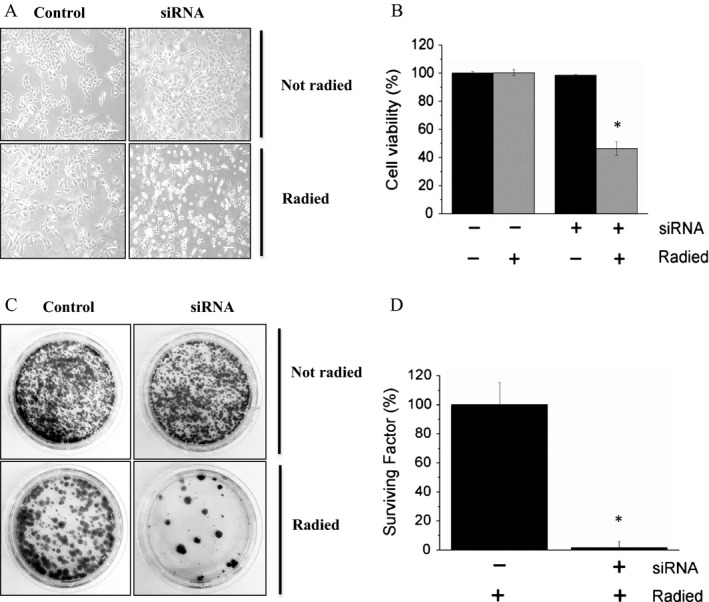
LMW‐PTP knockdown sensitizes melanoma cells to radiotherapy. (A) Images of parental and melanoma cells silenced for LMW‐PTP exposed to radiation (2 Gy). Images were obtained using a NIKON Eclipse TS100 contrast phase microscope (magnification 10×, scale bar = 100 *μ*m). (B) cell viability of radied cells determined using MTT assay. *P < 0.05. (C) Colony assay carried out after irradiation (2 Gy). (D) determination of surviving factor. After 10 days, colonies were stained using crystal violet and then counted to calculate the surviving factor. *P < 0.05

In light of the above results, we investigated whether by suppressing LMW‐PTP, it was possible to increase the sensitivity of melanoma cells to radiotherapy. Images obtained by contrast phase microscope show that only silenced cells exposed to 2 Gy radiation are rounded, or damaged (Fig. 2A). In keeping with this finding, MTT test confirmed that the vitality of silenced cells is dramatically lower to that of parental cells exposed to the same dose radiation (Fig. 2B). Finally, we observed that silenced cells exposed to radiations show a limited regenerative capability respect to control cells (Fig. 2C and D). In summary, our results demonstrate that LMW‐PTP suppression makes melanoma cells more sensitive to radiotherapy.

### Morin strongly enhances the cytotoxic activity of dacarbazine

**Figure 3:**
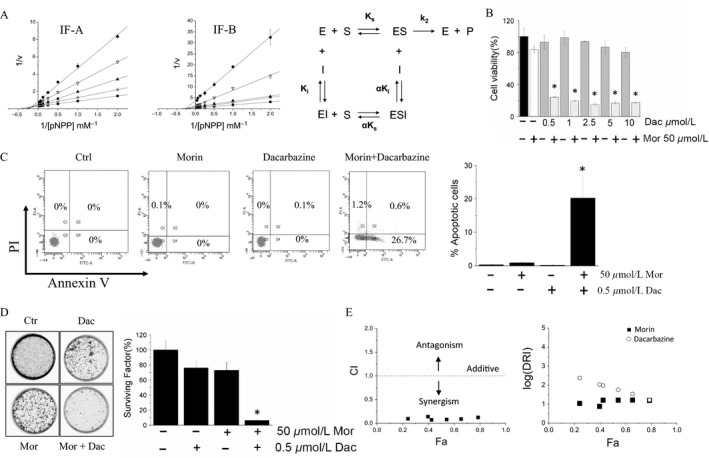
Morin enhances the cytotoxicity of dacarbazine. Double reciprocal plot obtained using recombinant isoform A (IF‐A) (A), and isoform B (IF‐B) of LMW‐PTP (B). (C) scheme of noncompetitive mixed type inhibition. (D) cell viability assay. A375 cells were treated with Morin alone (empty bar), increasing dacarbazine concentration (dark gray bars), or combination of Morin and dacarbazine (gray bars) for 48 h. All data were normalized with respect to control (black bar). Data represent the mean value ± SEM (*n* = 4). *P < 0.05. (E and F) evaluation of apoptosis. After 24‐h incubation, cells were stained with Annexin V/Pi method and analyzed by flow cytometry. For each test, 10,000 events were analyzed. Data reported in the graphic on the right represent the mean values ± SEM determined from three independent experiments (*n* = 3). (G and H) colony assay. Data reported in the figure represent the mean values ± SEM (*n* = 3). *P < 0.05. (I and L) analysis of the synergism between Morin and dacarbazine. Cell viability was assessed using the MTT test. Data obtained were analyzed using the CompuSyn software, considering that the combinations of drugs are in nonconstant ratio. Fa‐CI plot (Chou‐Talalay plot) (left); Fa‐DRI plot (Chou‐Martin plot) (right). CI < 1, synergism; CI = 1, additive; CI > 1, antagonism. DRI > 1, reduced dose and toxicity.

We evaluated whether using LMW‐PTP inhibitors, it was possible to reproduce, in vivo, the same effects as the LMW‐PTP silencing. To this aim, we selected Morin, a polyphenol that behaves as a mixed type noncompetitive inhibitor of LMW‐PTP, showing a Ki value in the micromolar range (Fig. 3A–C). We found that Morin, per sè, is not toxic but is able to enhance cytotoxic activity of dacarbazine (Fig. 3D–F). Furthermore, we observed that treatment of A375 cells with the combination Morin‐dacarbazine strongly inhibits renewal ability of cancer cells (Fig. 3G and H). To evaluate whether Morin synergizes with dacarbazine, we used the method proposed by T‐C Chou 14. The results show that 4 h preincubation with Morin followed by 48 h treatment with dacarbazine leads to a strong synergistic antiproliferative activity, as confirmed by low values of CI (the values are comprised between 0.071 and 0.14) (Fig. 3I and L), thereby confirming that Morin and dacarbazine synergize to induce melanoma cells death.

### Morin sensitizes A375 cells toward different chemotherapeutic agents

**Figure 4:**
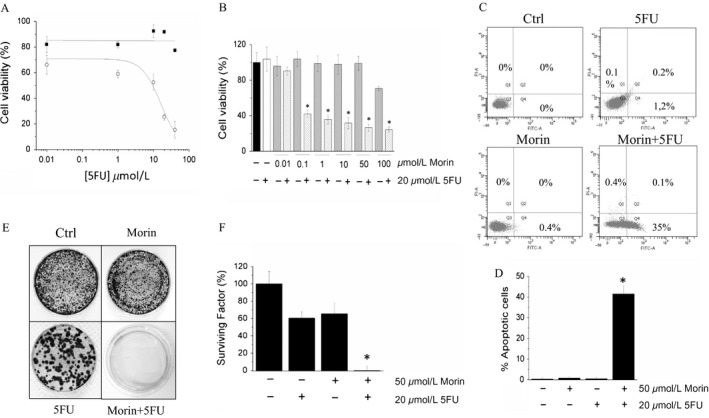
Morin enhances 5‐FU cytotoxicity. (A) determination of the IC_50_ value of 5‐FU for A375 cells, in the presence (open circles), or in the absence (black square) of 50 *μ*mol/L Morin. Cells viability was measured by MTT test. Data reported in the figure represent the mean values ± SEM (*n* = 3). (B) evaluation of A375 cell viability using increasing Morin concentration, and a fixed 5‐FU concentration. Experimental conditions are the same as in the panel A. *P < 0.05. (C and D) quantification of apoptotic events in A375 cells treated with Morin, 5‐FU, or a combination of both. Data reported in the graphic (bottom), represent the mean values ± SEM calculated from data obtained from three independent experiments (*n* = 3). *P < 0.05. (E and F) colony assay. Data represent the mean values ± SEM (*n* = 3). *P < 0.05.

We next investigated whether Morin could improve the effectiveness of other anticancer drugs such as the 5‐FU. We found that treatment with Morin strongly decreases the IC_50_ of 5‐FU for A375 cancer cells (Fig. 4A), in that Morin is already active as sensitizing agent at 0.1 *μ*mol/L concentration (Fig. 4B). From mechanistic point of view, we found that treatment with Morin is accompanied by a decrease in Bcl2 expression levels, by an increases in Bim (Fig. 1E), and the activation of caspase 3. Interestingly, a boost of apoptotic events was observed only in A375 cells treated with Morin/5‐FU combination, but not in cells treated with Morin alone (Fig. 4C and D). Finally, we showed that only co‐treatment with Morin and 5‐FU strongly impairs the colony forming ability of melanoma cells (Fig. 4E and F). In conclusion, our results demonstrate that Morin improves the effectiveness of 5‐FU.

### Co‐treatment with Morin and 5‐FU does not impair cell viability of noncancer cells

Results reported above demonstrate that Morin could be used as sensitizing agent to improve the effectiveness of anticancer drugs. However, we had to establish whether treatment with Morin could damage on noncancerous cells. To answer this question, different tests were carried out using noncancerous cells. No cytotoxic effects were detected treating differentiated C2C12 cells, Human Dermal Fibroblasts (HDF), and MCF10A human breast cells with Morin alone, or with the combination Morin‐5‐FU (Figure S2A–C). These results suggest that Morin sensitizes cancer cells to apoptosis without, however, affecting viability of normal cells.

### Morin induces a proteasome‐dependent LMW‐PTP degradation

**Figure 5:**
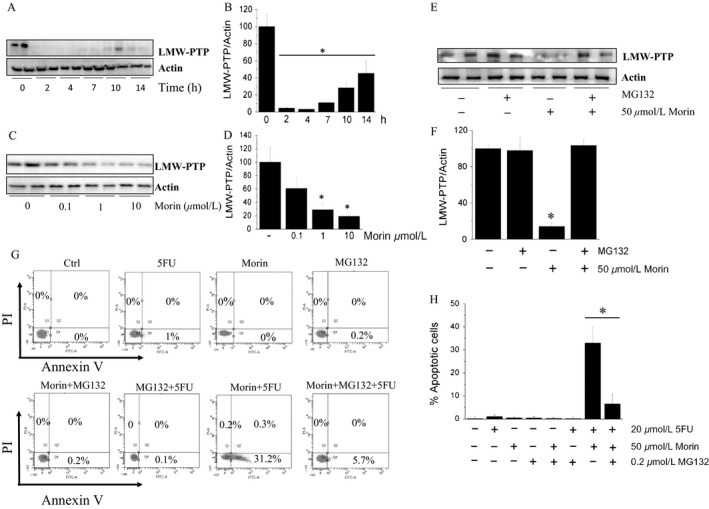
Morin induces LMW‐PTP degradation in A375 cells. (A and B) Time course analysis of LMW‐PTP expression levels in A375 cells treated with 50 *μ*mol/L Morin. (C and D) effect of different Morin concentrations on LMW‐PTP expression levels. Data represent the mean values ± SEM (*n* = 3). *P < 0.05. (E and F) MG132 inhibits LMW‐PTP degradation induced by Morin. A375 cells were preincubated for 1 h in the presence of 10 *μ*mol/L MG132 before treatment with 50 *μ*mol/L Morin. After 4 h, cell extracts were analyzed by western blot. Data reported in the figure (bottom) represent the mean values ± SEM (*n* = 2). *P < 0.05. (G and H) evaluation of apoptosis after 24 h incubation. Results reported in the figure represent the mean values ± SEM obtained from two independent experiments (*n* = 2). For each experiment, 10,000 events were acquired. *P < 0.05.

To gain insight into the mechanism of action of Morin, we evaluated whether it modulates LMW‐PTP expression. We found that treating A375 cells with Morin triggers a transient downregulation of LMW‐PTP levels (Fig. 5A and B). This effect is strictly dose dependent, and a significant decrease in LMW‐PTP levels was observed already using 1 *μ*mol/L Morin concentration (Fig. 5C and D). Interestingly, we found that the pre‐incubation of A375 cells with MG132 prevents LMW‐PTP downregulation (Fig. 5E and F), and almost completely abrogates the sensitizing effect of Morin (Fig. 5G and H), thereby confirming the existence of a strict correlation between LMW‐PTP downregulation and the increase in sensitivity of A375 cells to apoptosis.

Next, in order to evaluate the specificity of Morin in inducing LMW‐PTP degradation, we monitored also the expression levels of PTP1B and SHP2, two other phosphatases that are clearly involved in tumor progression 21, 22. We found that levels of PTP1B and SHP2 do not change after treatment with Morin (Figure S3A–C). In addition, SDS‐PAGE analysis of whole sample extracts shows that there are no differences between protein profiles of untreated cells with respect to that of cells treated with Morin (Figure S3D). Together, these results suggest that Morin triggers degradation of specific proteins, but does not causes a massive and nonspecific degradation of cellular proteins.

### Morin treatment increases sensitivity of A375 cells to radiotherapy

**Figure 6:**
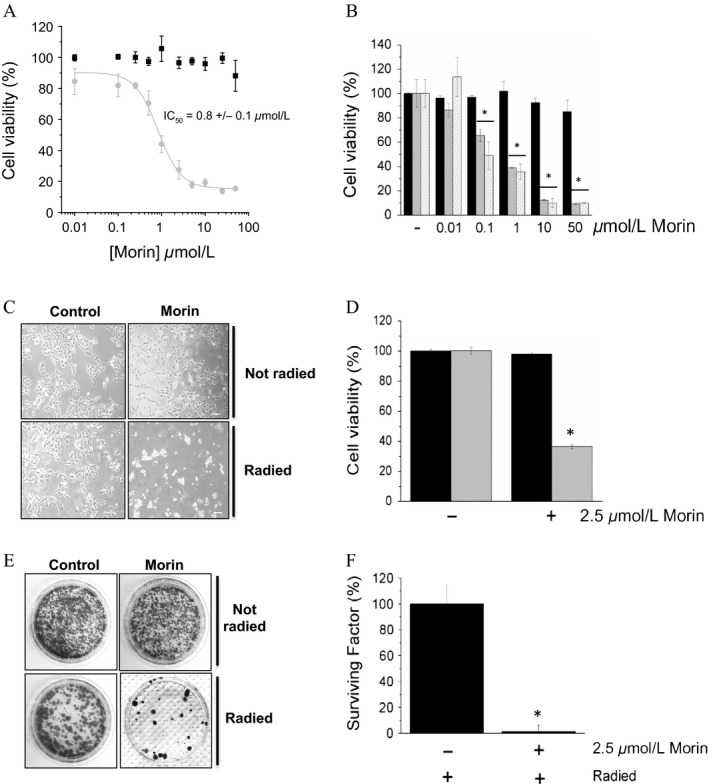
Morin sensitizes melanoma cells to radiotherapy. (A) Cell viability of A375 cells preincubated with increasing Morin concentrations (grey circles), or not (black squares), exposed to 4 Gy radiation evaluated by MTT assay. Data represent the mean values ± SEM (*n* = 4). (B) cell viability of cells treated with 50 *μ*mol/L of Morin and exposed to 1 (gray bar) or 2 Gy (dashed bar) radiation. Data reported in the figure represent the mean values ± SEM (*n* = 4). *P < 0.05. (C) effects of radiations (2 Gy) on A375 cells pretreated or not with 2.5 *μ*mol/L Morin. Cell images were obtained using a contrast phase microscope (C, magnification 10×, scale bar = 100 *μ*m). (D) Cell viability of radied cells was determined using MTT assay. Data reported in the figure represent the mean values ± SEM (*n* = 3). *P < 0.05. (E) colony assay; (F) determination of surviving factor. Data reported in the graphic represent the mean values ± SEM (*n* = 3). *P < 0.05.

Next, we investigated whether Morin is able to increase the sensitivity of A375 cancer cells to radiotherapy. MTT test reveals that pretreatment with Morin strongly increases radio‐sensitivity of A375 cells in a dose‐dependent manner, being 0.8 *μ*mol/L the IC_50_ value of Morin (Fig. 6A). By treating melanoma cells with a lower radiation dose, 1 or 2 Gy, we obtained similar results (Fig. 6B). Noteworthy, combining Morin pretreatment with radiation (2 Gy), it is possible to strongly reduce both cell viability and colony formation ability of melanoma cells (Fig. 6C and D). Together, these results confirm that Morin is able to enhance the effectiveness of radiotherapy (Fig. 6E and F).

### Morin treatment, and LMW‐PTP silencing sensitize PC3 cancer cells toward docetaxel, and radiotherapy

In line with the above results, we found that LMW‐PTP silencing (Figure S4A and B) enhances the sensitivity of PC3 prostate cancer cells to docetaxel (Figure S4C and D) and impairs their self‐renewal ability (Figure S4E and F). In addition, we found that, in PC3 cells, Morin induces a transient downregulation of the LMW‐PTP levels (Figure S5A and B), and that its combination with docetaxel reduces cancer cells viability (Figure S5C) and enhances apoptosis rate (Figure S5D and E). This combination also increases the effectiveness of radiotherapy (Figure S5F), almost to the complete inhibition of PC3 cells ability to form new colonies (Figure S5G and H). These results confirm that LMW‐PTP knockdown enhances sensitivity to apoptosis of PC3 cells, and that pretreating PC3 cells with Morin it is possible to reproduce the same results obtained by LMW‐PTP silencing.

## Discussion

Statistics on cancer incidence and survival data revealed that melanoma represent one of the deadliest tumors that affects man. This is due, in part, to the ability of melanoma cells to spread and metastatize, and, in part, to intrinsic resistance of melanoma cells to traditional anticancer therapies.

Studies conducted in the past demonstrate that several factors contribute to resistance of melanoma cancer cells to anticancer therapies, including constitutive activation of pro‐survival pathways, overexpression of DNA repairing enzymes, of drug extrusion pumps, and of antiapoptotic proteins 6.

Recent studies identify the LMW‐PTP as a new negative effector of apoptotic pathway. It has been demonstrated that this enzyme, generally overexpressed in cancer cells 8, contributes to improve resistance of cancer cells toward cytotoxic agents 9, 11, 12, thereby favoring cancer cell survival and proliferation. To date, the role of this protein in skin cancer and melanoma remains to be clarified.

Data reported in this study demonstrate that the expression of high LMW‐PTP levels is functional in making melanoma cells more invasive and resistant to apoptosis. Tests carried out using RNA interference confirm this hypothesis. Indeed, we found that the knockdown of LMW‐PTP affects cell adhesion and invasiveness and is accompanied with a strong decrease in Bcl2 levels, an increase in Bim expression, and with the activation of caspase 3. In the same moment, we observed that silenced cells become more sensible to dacarbazine, 5‐FU, and radiotherapy. Taken together, these findings suggest that LMW‐PTP regulates cell morphology, motility, invasiveness, and sensitivity to apoptosis. Some of these effects were not completely unexpected. Indeed, previous studies demonstrated that LMW‐PTP targets and inactivates p190Rho‐GAP, thereby promoting activation of Rho, a GTPase involved in regulation of cell migration 23. Thus, we speculate that the changes on cell morphology and motility observed in melanoma cells after LMW‐PTP silencing could be due to constitutive Rho inactivation. However, whether, and how Rho inactivation can lead to improve sensitivity to apoptosis of cancer cells remain to be clarified. A recent study demonstrates that Rho overexpression enhances synthesis of ABC transporters and reduces the sensitivity of colon cancer cells to apoptosis induced by treatment with irinotecan. Conversely, Rho silencing suppresses the expression of Bcl‐xl and Bcl2 and promotes the expression of Bax 24. In light of this findings, we hypothesize that LMW‐PTP‐mediated Rho activation could be the mechanism that drives the conversion of melanoma cells toward an aggressive and drug‐resistant phenotype. Further studies will be needed to clarify the role of Rho, and the mechanisms by which Rho contributes to regulate expression of both anti‐ and pro‐apoptotic proteins in melanoma cells.

Although many studies have confirmed the importance of the PTPases as pharmacological targets, for long time these have been considered “undruggable enzymes,” especially because of the difficulties encountered in generating sufficiently specific inhibitors for members of this family 25.

Data reported in this study for the first time demonstrate that by treating melanoma cells with Morin, it is possible to reproduce in vivo all effects obtained with LMW‐PTP silencing. Morin is a polyphenol that shows peculiar properties: in vitro*,* it behaves as a noncompetitive inhibitor of LMW‐PTP, whereas, in vivo, it triggers the specific and transient degradation of LMW‐PTP, through a proteasome‐dependent mechanism. In addition, we found that treatment with Morin is associated with the reduction in Bcl2 levels, the increase in Bim expression, and with the activation of caspase 3. Moreover, we demonstrated that Morin synergizes with dacarbazine and 5‐FU and potentiates the effectiveness of radiotherapy. Another interesting aspect of Morin is its probed specificity against cancer cells. Indeed, we observed that combined therapy (Morin‐chemotherapy, or Morin‐radiotherapy) impairs viability of cancer cells, but does not affect survival of noncancerous cells. This is due to the ability of Morin to triggers degradation of LMW‐PTP, which is usually overexpressed in cancer cells, such as melanoma and PC3 cancer cells, but is quite undetectable in noncancerous cells, such as fibroblast and muscle cells. This finding suggests that Morin could be used as an innovative weapon to selectively enhance the effectiveness of anticancer drugs against cancer cells, thereby improving clinical outcome, without the risk to induce severe side effects. In keeping with this hypothesis, we demonstrated also that combined treatment is more effective than traditional anticancer therapies in inhibiting clonogenicity of melanoma and PC3 cancer cells. Together, data reported in this study suggest that Morin acts with a double mechanism: In a first phase, it enhances the effectiveness of traditional anticancer therapies, promoting the killing of proliferating cancer cells; however, at long‐term period, it inhibits proliferation of cancer cells characterized by high clonogenic power, thereby reducing the risk of relapses after treatment.

In summary, results shown in this study demonstrate, for the first time, that in melanoma and in PC3 cells LMW‐PTP is directly involved in the control of apoptosis and that by regulating its expression levels it is possible to improve the sensitivity of cancer cells toward anticancer therapies. Moreover, we identify the polyphenol Morin as a LMW‐PTP inhibitor able to induce transient downregulation of this enzyme and to reproduce all effect obtained by gene silencing. These results could have important implications in the field of anticancer therapies because they demonstrate the importance of this enzyme as a target to control melanoma cell growth and highlight the possibility to use LMW‐PTP inhibitors to develop new adjuvant‐based anticancer therapies beneficial for the treatment of metastatic melanoma and other kind of drug‐resistant cancers.

## Conflict of Interest

The authors declare no potential conflicts of interest.

## Supporting information


**Figure S1.** LMW‐PTP is highly expressed in melanoma, and its down‐regulation affects morphology, adhesion and invasiveness of A375 cells.

**Figure S2.** Effects of combined treatment with Morin and 5‐FU on viability of non‐cancerous cells.

**Figure S3.** Morin does not induces degradation of PTP1B and SHP2 phosphatases.

**Figure S4.** LMW‐PTP knockdown improves sensitivity of PC3 cells to docetaxel, and impairs their self‐renewal ability.

**Figure S5.** Morin enhances sensitivity of PC3 cells toward docetaxel, and impairs their self‐renewal ability
