# Supplementary figures and images for: Targeting LMW‐PTP to sensitize melanoma cancer cells toward chemo‐ and radiotherapy
Source: Cancer Med. 2018 Mar 24;7(5):1933–43. doi: 10.1002/cam4.1435 (PMC5943542; doi:10.1002/cam4.1435)

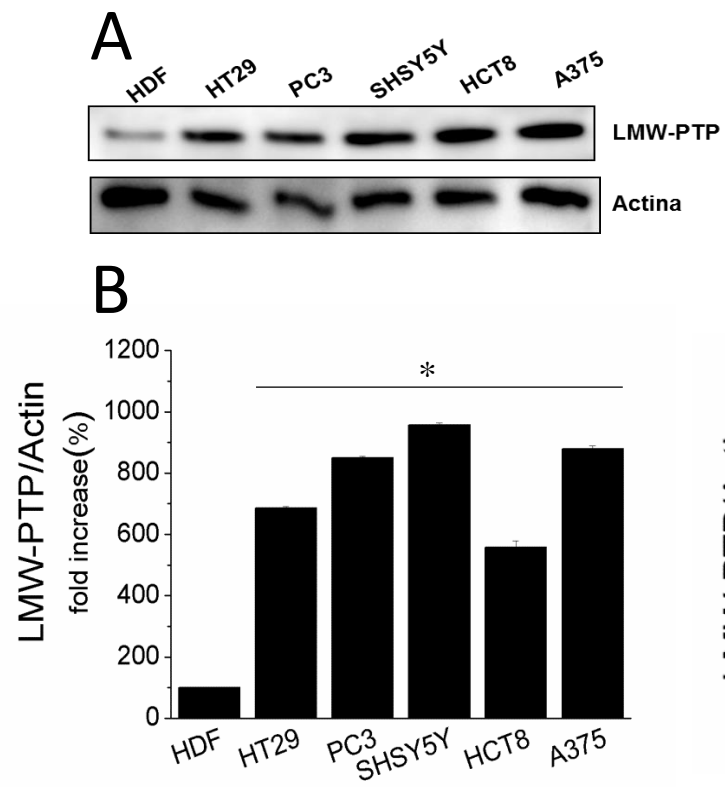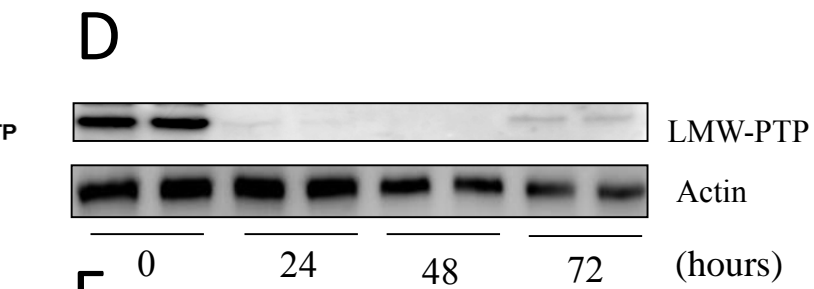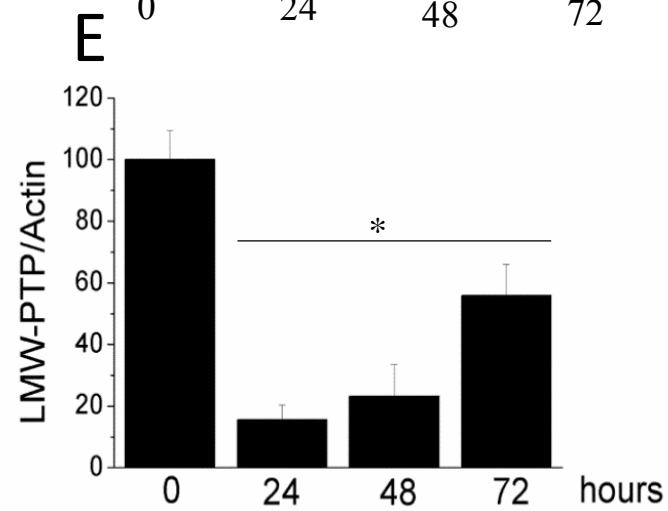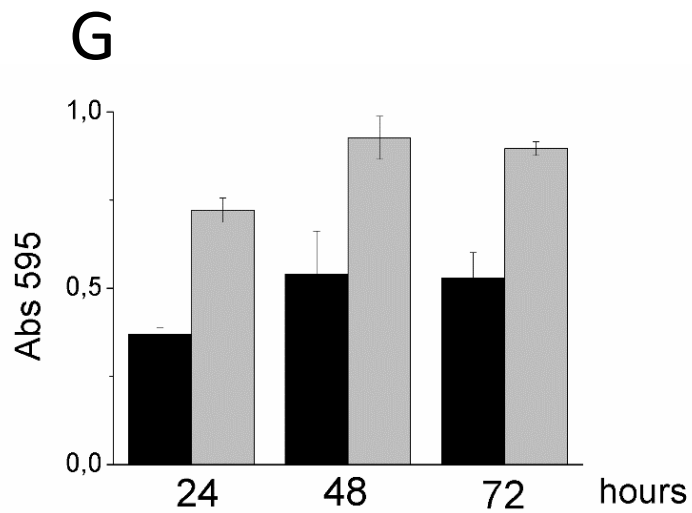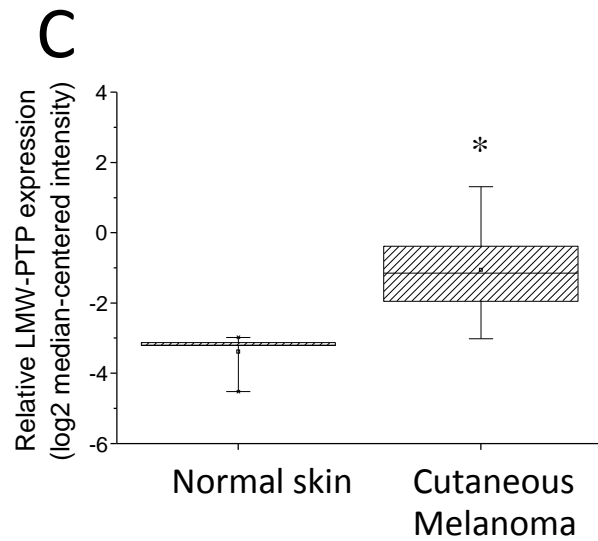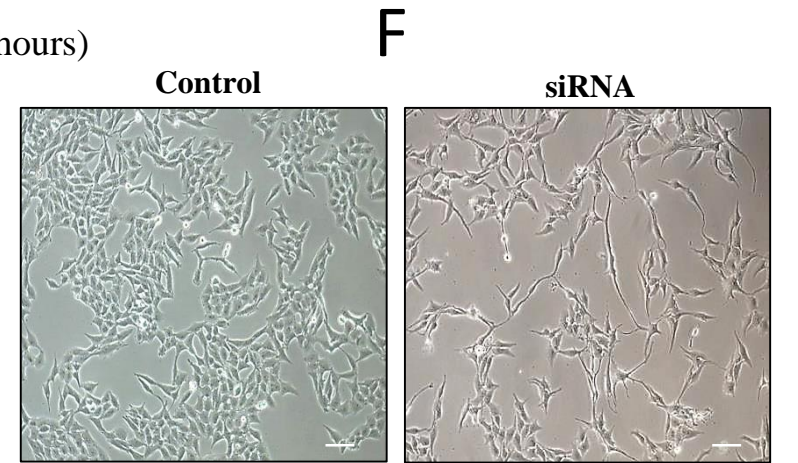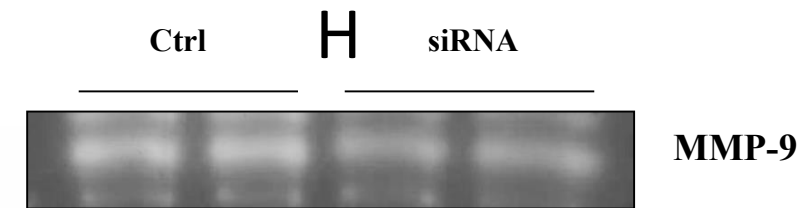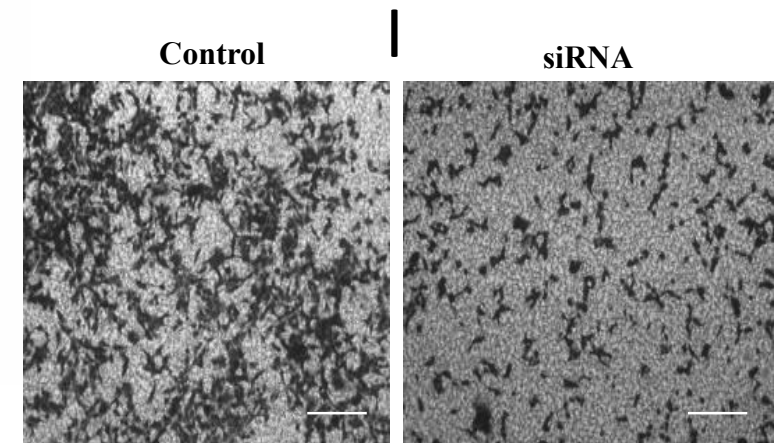

Supplement: Supplementary file 1 — Figure S1. LMW‐PTP is highly expressed in melanoma, and its down‐regulation affects morphology, adhesion and invasiveness of A375 cells. [file CAM4-7-1933-s001.pdf]

0%

0%

0%

0%

0%

0%

0%

0%

0%

0%

0%

0%

C

Ctrl

5FU

Morin

Morin+5FU


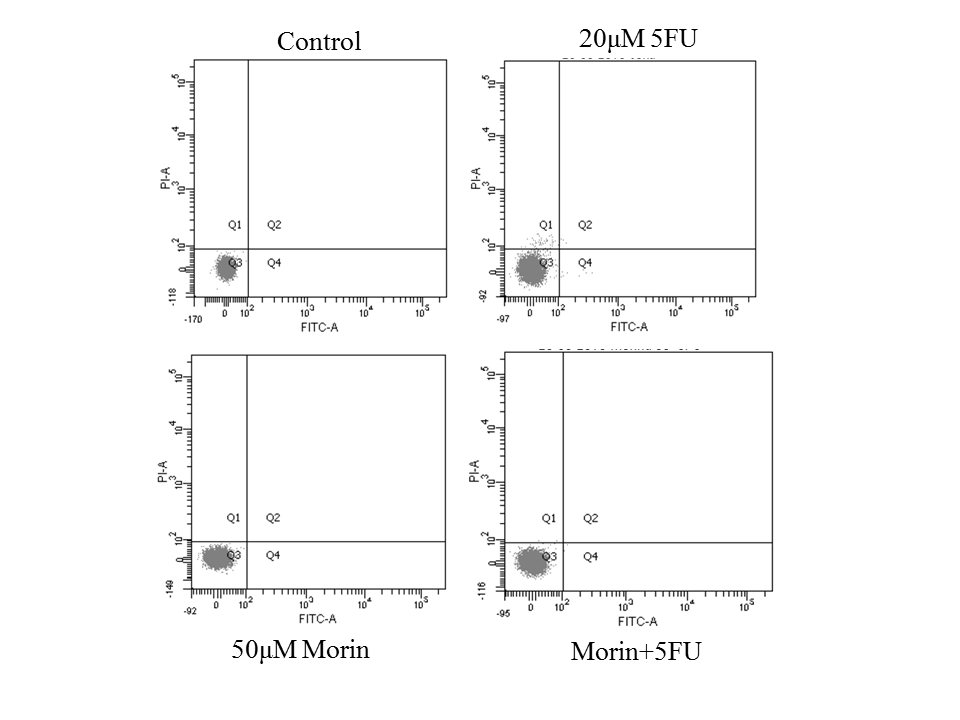

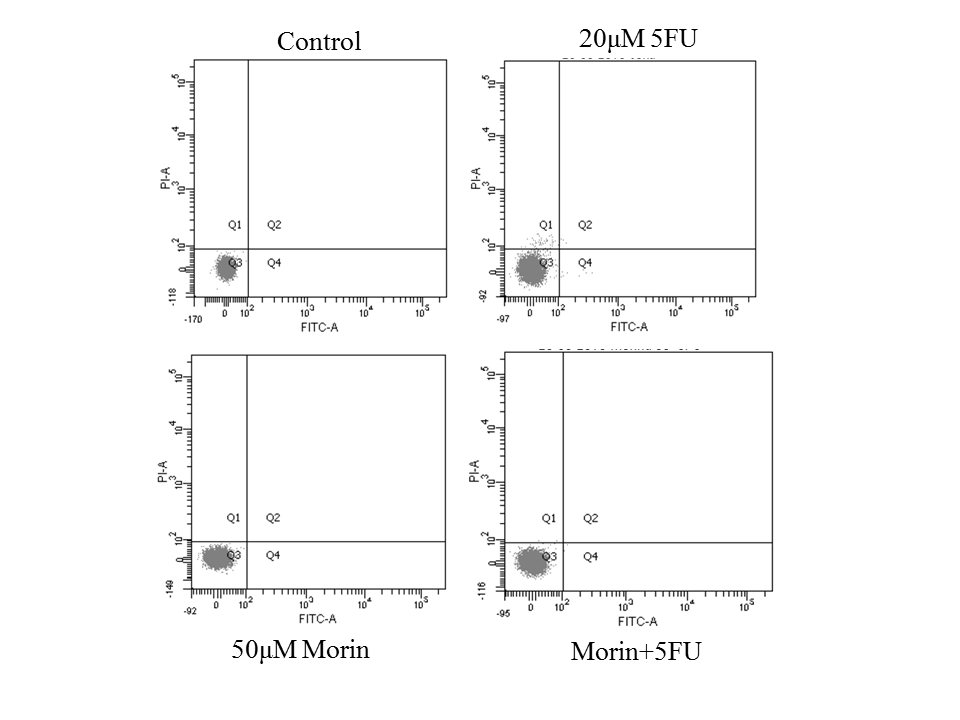

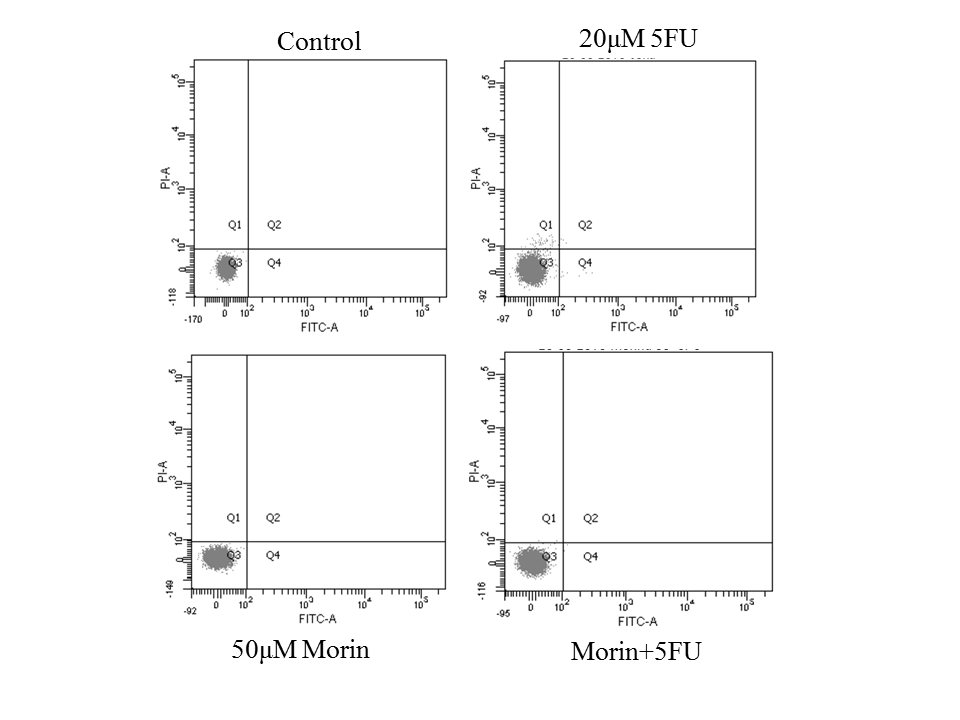

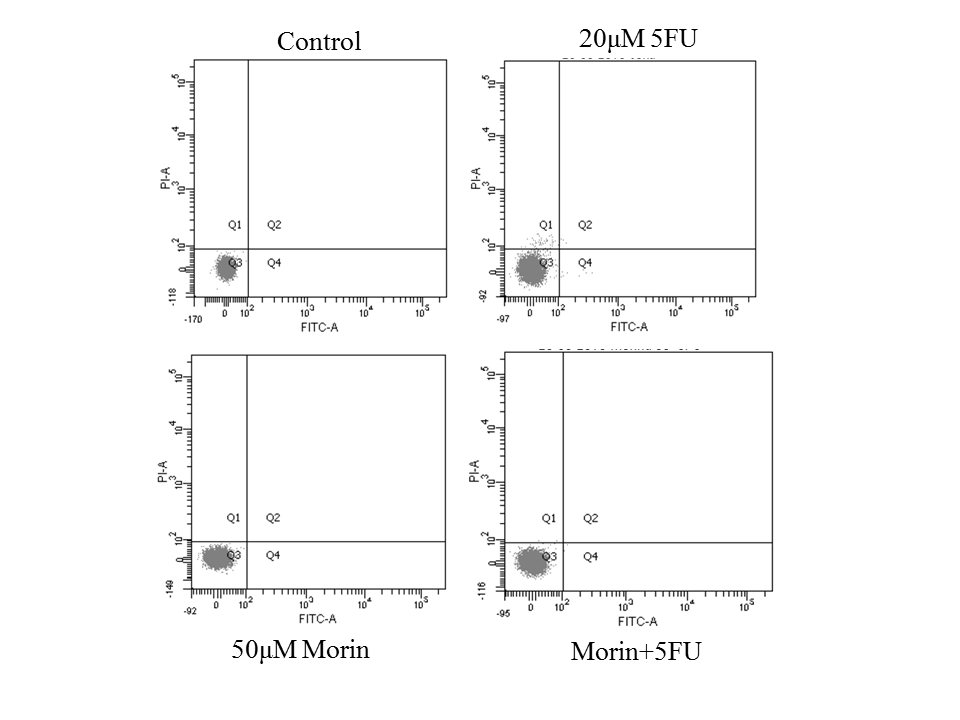


Annexin V

PI

B

A

PI

Annexin V

MCF10A

HDF

0%

0%

0%

0%

0%

0%

0%

0%

0%

0%

0%

Ctrl


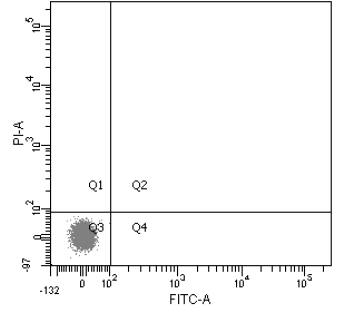


0%

0%

0%


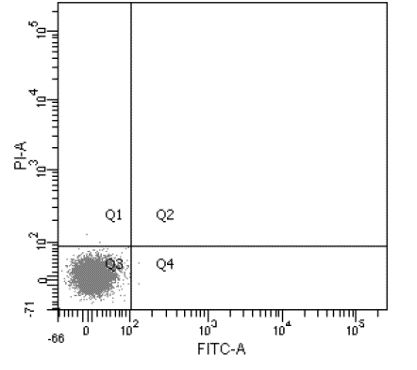


5FU

0,3%

0%

0%


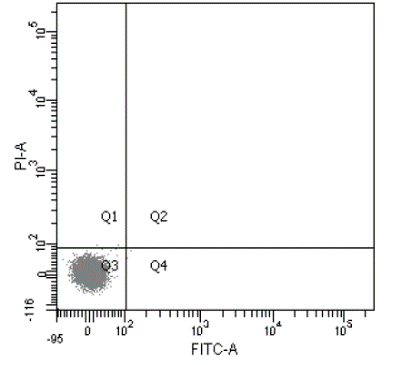


0%

0%

0,1%

Morin


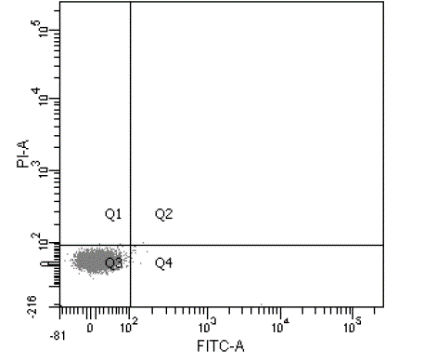


0,2%

Morin+5FU

0%

0%

0%

Supplement: Supplementary file 2 — Figure S2. Effects of combined treatment with Morin and 5‐FU on viability of non‐cancerous cells. [file CAM4-7-1933-s002.docx]

A


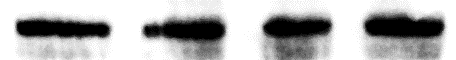

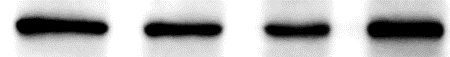

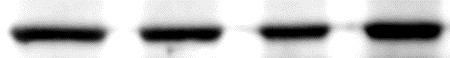


SHP2

PTP1B

Actin

Time (h)

0

2

4

6

B

C





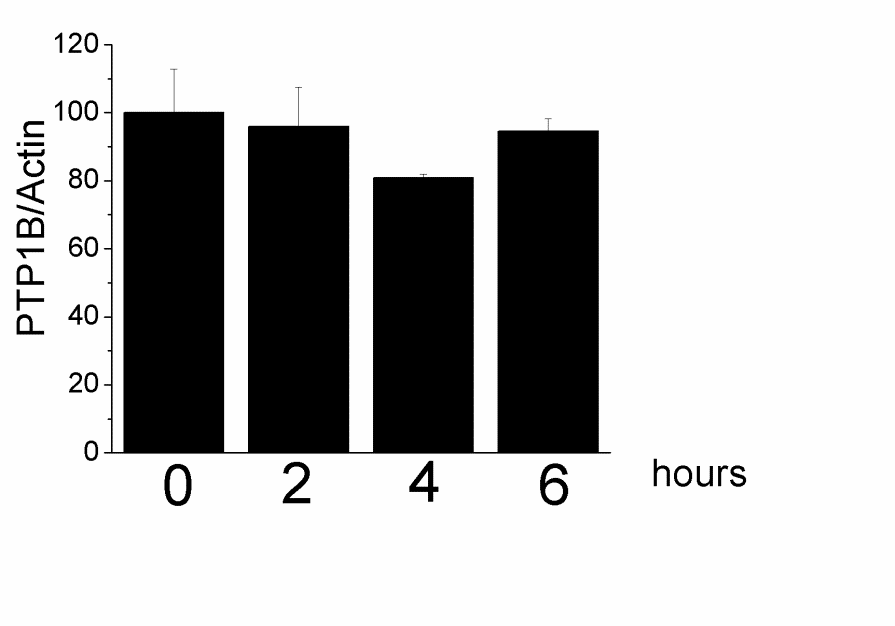


0

2

4

6

6

4

2

0

D


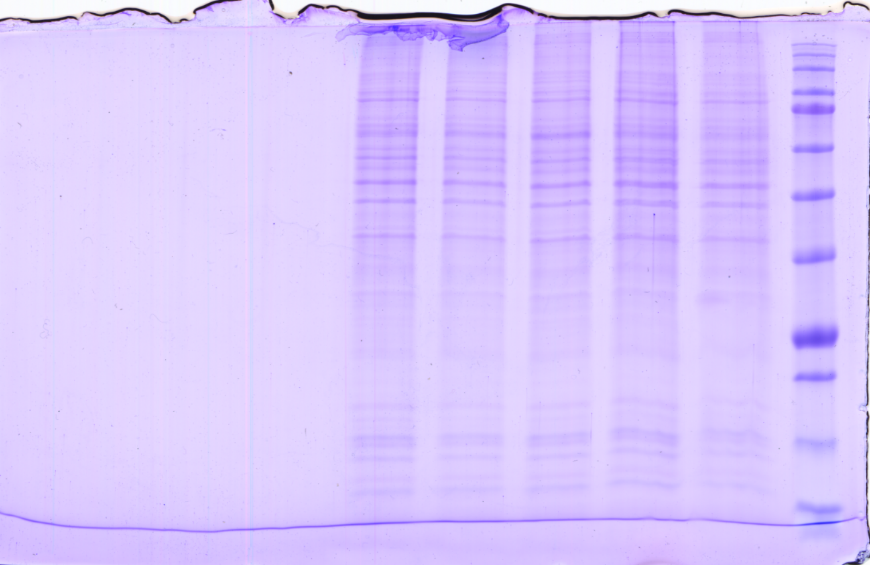


2.5

5.0

10

50

0

St

Morin (μM)

Supplement: Supplementary file 3 — Figure S3. Morin does not induces degradation of PTP1B and SHP2 phosphatases. [file CAM4-7-1933-s003.docx]

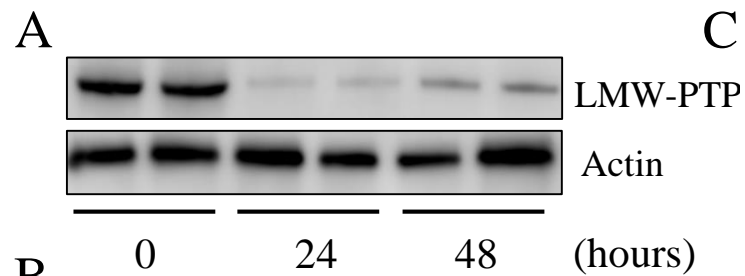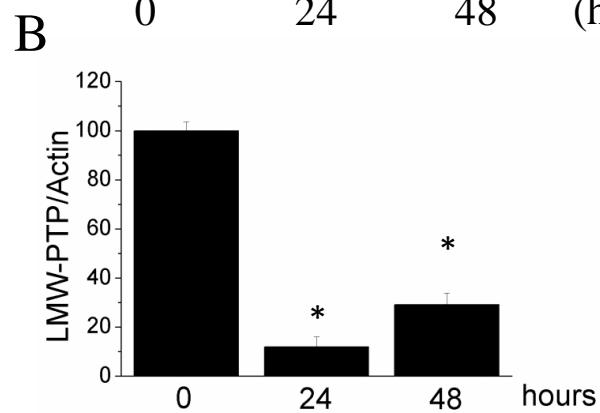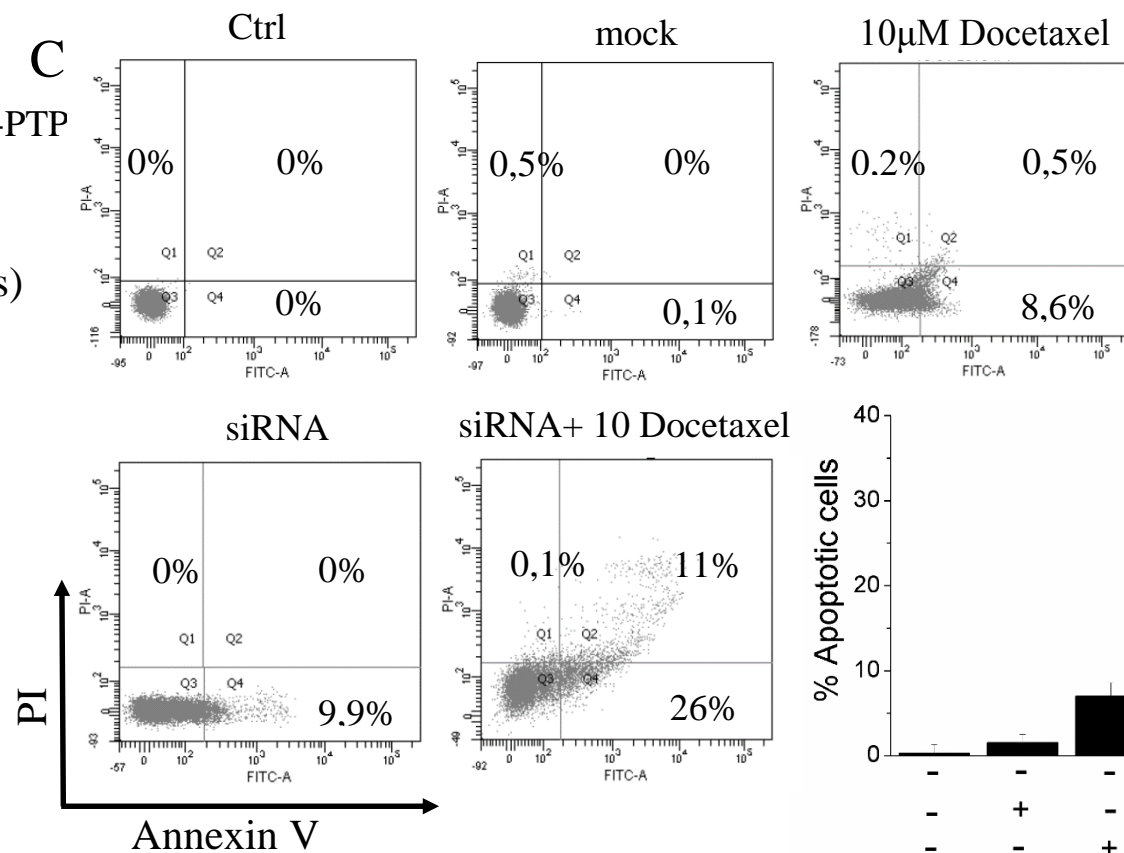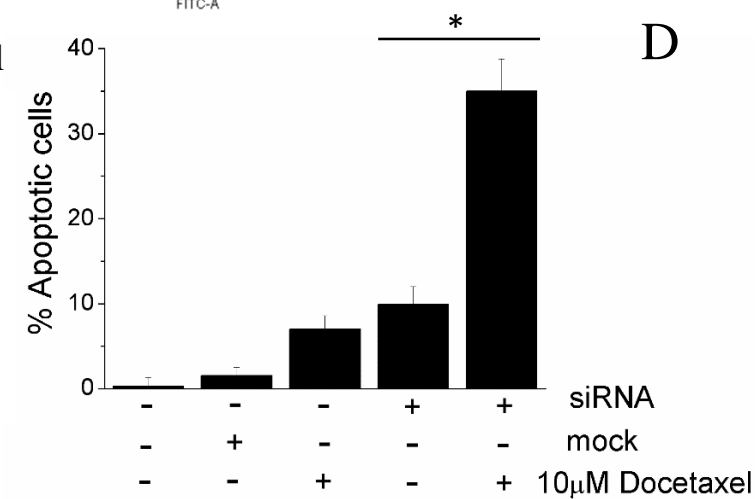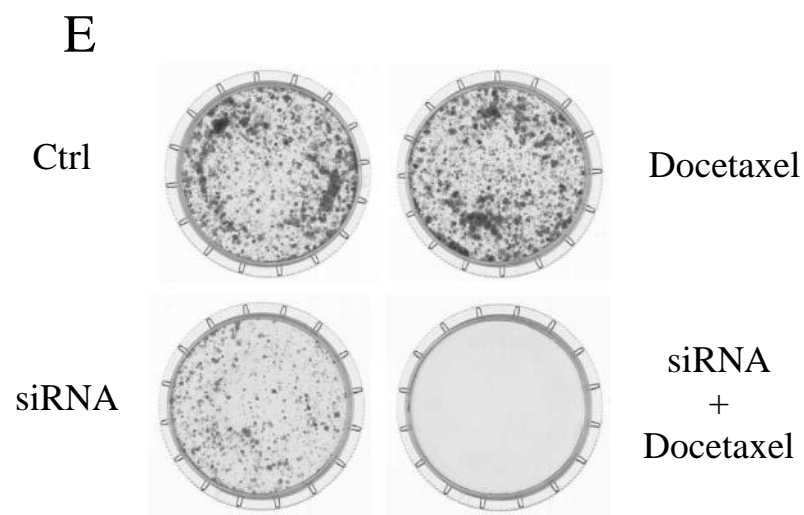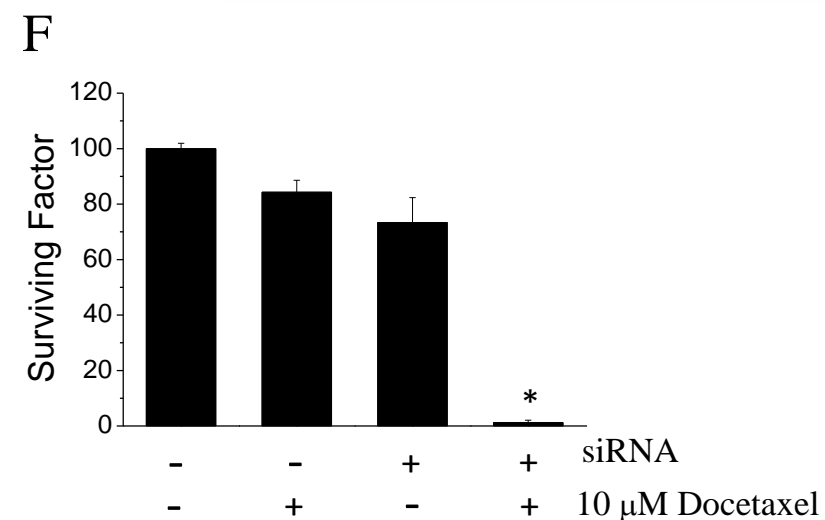

Supplement: Supplementary file 4 — Figure S4. LMW‐PTP knockdown improves sensitivity of PC3 cells to docetaxel, and impairs their self‐renewal ability. [file CAM4-7-1933-s004.pdf]
