# Supplementary material for: Targeting LMW‐PTP to sensitize melanoma cancer cells toward chemo‐ and radiotherapy
Source: Cancer Med. 2018 Mar 24;7(5):1933–43. doi: 10.1002/cam4.1435 (PMC5943542; doi:10.1002/cam4.1435)
Supplement: Supplementary file 5 — Figure S5. Morin enhances sensitivity of PC3 cells toward docetaxel, and impairs their self‐renewal ability [file CAM4-7-1933-s005.docx]

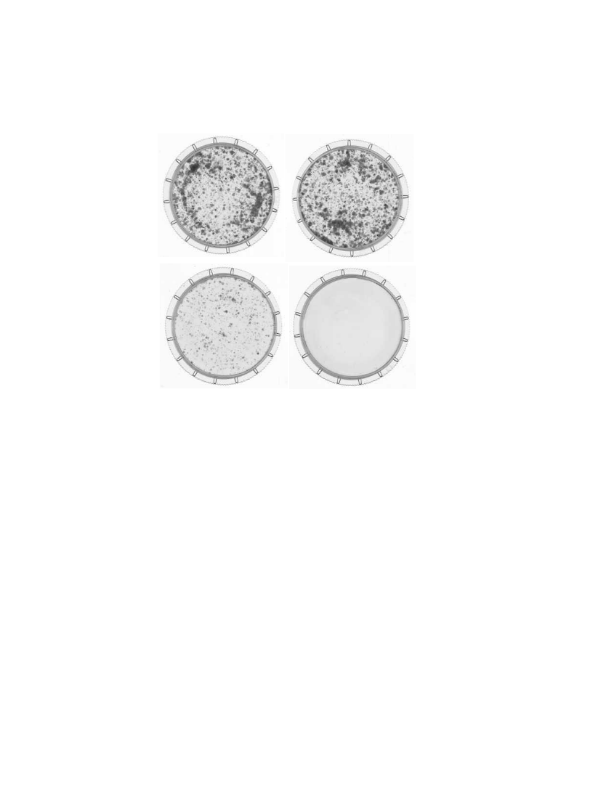


Ctrl

Docetaxel

Morin

Morin

+

Docetaxel

H

E

F

B


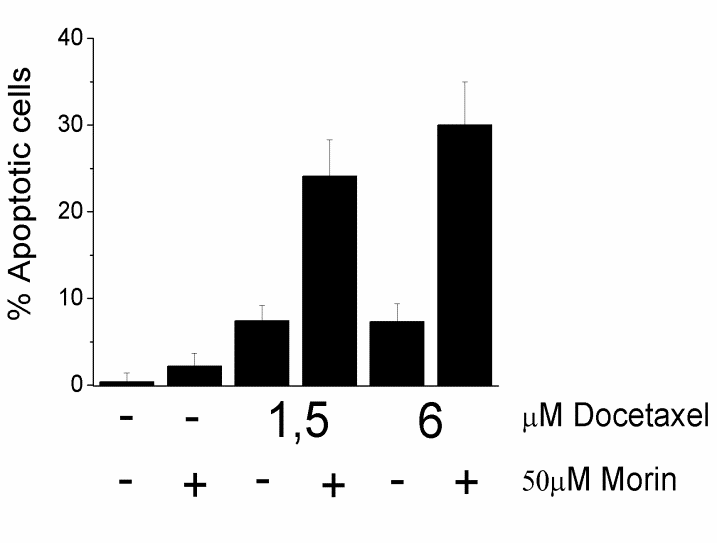


*

*


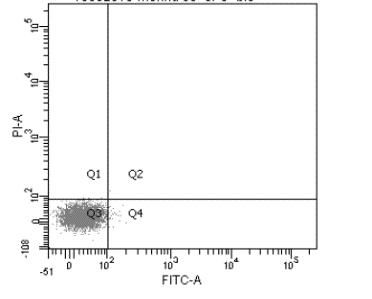


Morin

0%

0%

0,5%


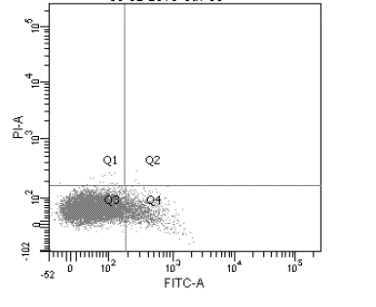


0,1%

0%

10,1%

1,5μM Doc


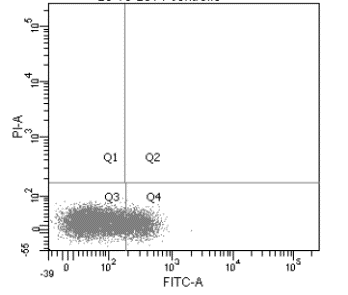


21,2%

0%

0%

1,5μM Doc+Morin


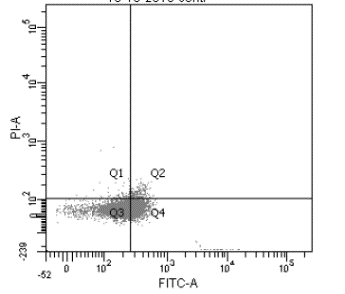


6μM Doc+Morin

0,5%

1,6%

34,4%


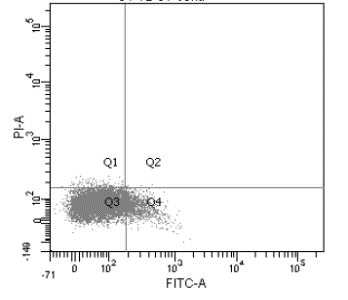


0,7%

0%

10,9%

6μM Doc


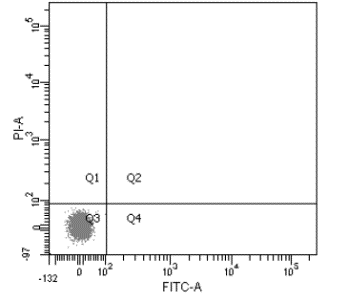


Ctrl

0%

0%

0%

Annexin V

PI

A

D


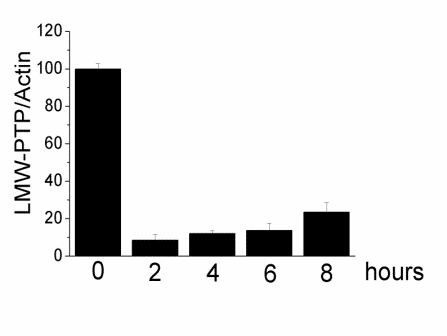


*


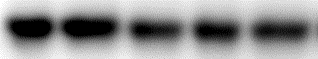

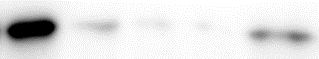


LMW-PTP

Actin

Time (h)

0

2

4

6

8

G


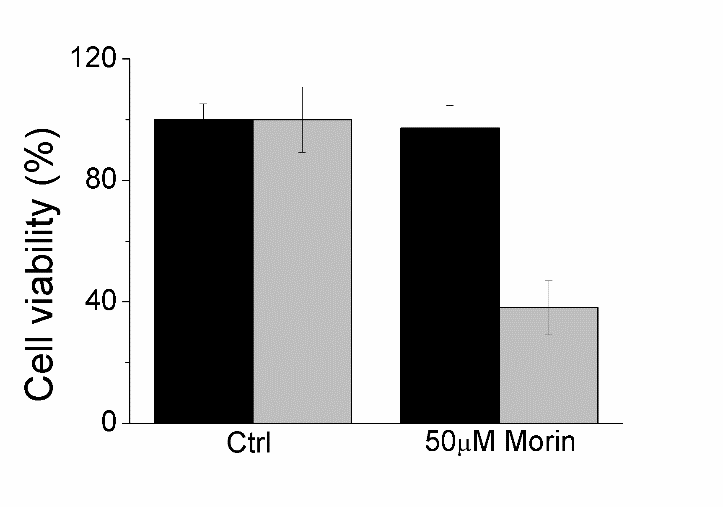


*

*

C

*

Annexin V

PI
